# PPARs as determinants of the estrogen receptor lineage: use of synthetic lethality for the treatment of estrogen receptor-negative breast cancer

**DOI:** 10.18632/oncotarget.17302

**Published:** 2017-04-20

**Authors:** Robert I. Glazer, Levy Kopelovich

**Affiliations:** ^1^ Department of Oncology, Georgetown University School of Medicine, and Lombardi Comprehensive Cancer Center, NW, Washington, D.C., USA; ^2^ Department of Medicine, Weill Cornell College of Medicine, New York, NY, USA

**Keywords:** PPARδ, PPARγ, ER

## Abstract

**The Dilemma:**

Estrogen receptora-negative (ER-) breast cancer lacks a specific critical target to control tumor progression.

**The Objective:**

To identify mechanisms that enable increased expression of the ER+ lineage in an otherwise ER- breast cancer.

**Preface:**

The nuclear receptor superfamily members PPARγ and PPARδ regulate gene expression associated with a multitude of pathways, including intermediary metabolism, angiogenesis, proliferation and inflammation (see reviews [1–3]). Recent developments using transgenic and knockout mice, as well as pharmacologic intervention with PPARγ and PPARδ agonists, have revealed a previously unknown relationship between PPARγ suppression and PPARδ activation that leads to the appearance of ER+ tumors, enabling a synthetic lethality approach by anti-ER therapy. The ability to selectively affect the ER+ lineage by modulating PPARγ and PPARδ activity represents a new clinical paradigm and opportunity to treat ER- cancer with PPARγ and PPARδ modulating agents, ultimately rendering them more responsive to adjuvant therapy.

## PRECLINICAL BACKGROUND

### PPARγ

#### Inhibition of PPARγ through a dominant-negative transgene or by pharmacologic intervention enables a transition from an ER^-^ to an ER^+^ lineage enrichment in breast cancer

**Figure 1:**
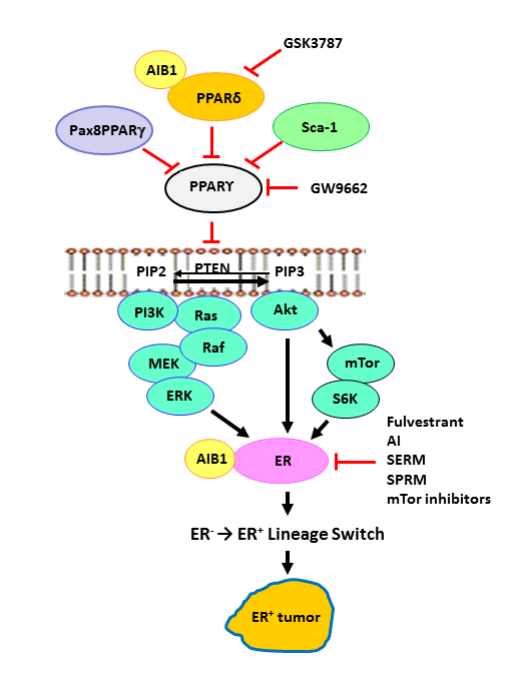
PPARs and the ER+ lineage Dominant-negative Pax8-PPARg, PPARd, Sca-1/Ly6a and PPARg inhibitor GW9662 each result in attenuation of the tumor suppressor effects of PPARg, eg. PTEN expression [5–7, 9, 19, 24], which was previously shown to occur transcriptionally [39]. Higher ratios of PPARd/PPARg promote the expansion of the ER^+^ progenitor lineage, leading to development of ER^+^ tumors. This paradigm suggests that negative regulation of PPARg or positive regulation of PPARd will enhance sensitivity to endocrine and targeted therapy by a mechanism analogous to synthetic lethality. GSK3787, PPARd inhibitor; GW9662, PPARg inhibitor; AI, aromatase inhibitors; SERM, selective ER modulators; SPRM, selective PR modulators.

The evidence: The role of PPARγ in lineage specification is often thought of in the context of its ability to regulate several tumor suppressor genes. Evidence to support a role for PPARγ in the development of the ER+ lineage was provided by transgenic mice expressing the fusion protein Pax8PPARγ, a dominant-negative form of PPARγ [4, 5], that is expressed in follicular thyroid cancer as a result of a t(2;3)(q13;p25) translocation between the paired-box transcription factor Pax8 and PPARγ [4]. Induction of mammary carcinogenesis in this transgenic model led to the appearance of ER^+^ tumors that were exquisitely sensitive to the ER antagonist fulvestrant [6] (Figure 1). These findings led us to determine whether the irreversible PPARγ antagonist GW9662 could act as a pharmacologic mimic of Pax8PPARγ and similarly induce the appearance of ER^+^ tumors in an otherwise ER- animal model. GW9662 did in fact replicate many of the phenotypic features of Pax8PPARγ transgenic mice and similarly rendered tumors sensitive to fulvestrant [7] (Figure 1). Thus, it was now possible to pharmacologically manipulate tumor lineage by inhibiting PPARγ, and in essence achieve a synthetic lethal effect [8] to endocrine therapy.

Since Pax8PPARγ induced a progenitor cell phenotype by PPARγ suppression, we examined if the converse would be true, viz. whether deficiency of the progenitor cell factor Stem Cell Antigen-1 (Sca-1/Ly6a) would upregulate the expression of PPARγ. Induction of mammary carcinogenesis in Sca-1 knockout mice led to a marked increase in PPARγ expression and to a synthetic lethal effect by the PPARγ agonist GW7845 [9].

Further insight into how PPAR could modulate the ER^+^ tumor lineage was suggested by the coactivator/corepressor dynamics of the ER [10]. PPARγ interferes with ER transactivation by binding to canonical ER response elements [11, 12] in a fashion similar to ER inhibition of PPAR response element (PPRE)-dependent transcription [13]. PPARγ and PPARδ have opposing actions either by direct competition [14], coactivator competition [15] and/or ligand-dependent activation and repression [16]. Additional studies using MMTV-AIB1 transgenic mice support this notion, where AIB1coactivator expression led to the development of ER+ tumors [17, 18]. This phenotype is similar to what we have recently reported for MMTV-PPARδ mice [19], and supports the concept that ligand-dependent recruitment of coactivators to PPARδ promotes ER^+^ progenitor cell expansion and oncogenesis by blocking the negative regulatory effects of PPARγ on this lineage (Figure 1). Interestingly, tumorigenesis in both AIB1 and PPARδ mice was dependent on mTOR activation downstream of phospholipid catabolism and an inflammatory phenotype [17], which may suggest a possible link between lipid biosynthesis, ER^+^ breast cancer and obesity, particularly in postmenopausal women [20].

### PPARδ

#### Overexpression of the PPARδ transgene in the presence of an agonist enables a transition from ER^-^ to an ER^+^ lineage enrichment in breast cancer

The evidence: PPARδ was shown to stimulate mitotic clonal expansion of progenitor cells more than a decade ago [21, 22]. It was conceivable, therefore, that the selective PPARδ agonist GW501516 [23] would act as a tumor promoter in mammary carcinogenesis, which proved to be the case [24] (reviewed in [2, 25]). Conversely, disruption of PPARδ expression reduced tumorigenesis in experimental breast cancer models [26]. It is interesting to note that 50% of invasive breast cancers expressed moderate to high levels of PPARδ protein [2] and that 65% of this type of breast cancer express increased PPARδ mRNA, whereas, the reverse is true for normal breast (www.oncomine.org;TCGA database). Thus, the majority of aggressive breast cancers would be expected to be responsive to a PPARδ agonist or PPARγ antagonist that could reverse the negative regulation by PPARγ on the ER^+^ lineage.

Etiological factors relevant to breast cancer that are directly relevant to pharmacological modulation of PPARs are obesity and inflammation [27]. These processes ultimately provide a milieu for the biosynthesis of endogenous PPARδ ligands [28], including arachidonic metabolites PGI_2_ [29] under the control of the PPRE-regulated gene Cox2/Pges2 [30], 15-HETE [31] and polyunsaturated fatty acids [32–34], under the control of PPAR-responsive genes. In this context, PPARγ activation by GW501516 increased arachidonic and linoleic acids in mammary tumors [35], and elicited an inflammatory gene signature in other animal models [36, 37], which are also consistent with PPARδ-mediated repression of PPARγ [14]. Additionally, metabolomic analysis of these mice revealed increased levels of lysophosphatidic acid and phosphatidic acid, both positive effectors of mTOR activity, and rendered PPARδ mice sensitive to the antitumor activity of the rapamycin analog everolimus [17].

## CLINICAL RELEVANCE

The ability of PPAR agonists and antagonists to modulate oncogenic signaling pathways provides a therapeutic paradigm through which presentation of the ER^+^ lineage is favored, leading to several important clinical strategies for the treatment of ER^-^ breast cancer.

First, PPARγ inhibition should enhance the appearance of the ER^+^ tumor lineage, enabling sensitivity to anti-ER therapy.

Second, PPARδ activation should similarly promote the development of ER^+^ oncogenesis through its negative regulatory effect on PPARγ, while simultaneously activating of mTor signaling.

Third, the opposing actions of PPARγ and PPARδ provide a framework to test this proposition, which would lead to a synthetic lethal approach to therapy, whereby phenotypically ER- tumors are rendered ER^+^ by treatment with a PPARγ antagonist and/or a PPARδ agonist to enable targeting the ER with endocrine therapy.

Fourth, Potential candidate populations might include patients with clinically overt triple-negative breast cancer or luminal B breast cancer with low ER expression.

## TREATMENT SCHEDULING

We envision a therapeutic paradigm where patients would receive treatment with a PPARγ antagonist and/or a PPARδ agonist to activate the ER^+^ lineage simultaneously with chemotherapy plus an ER inhibitor such as fulvestrant or an aromatase inhibitor. Since we have also observed the dependence of PPAR-mediated tumorigenesis on mTOR signaling [19], combining endocrine therapy with a rapamycin analog should further block tumor progression as found in experimental models [38].
